# COVID-19 psychological impact in patients with depressive disorder: Differences based on their age

**DOI:** 10.1192/j.eurpsy.2021.1790

**Published:** 2021-08-13

**Authors:** E. Martín Gil, M. Valtueña-García, L. González-Blanco, F. Dal Santo, C. Moya-Lacasa, C. Álvarez Vázquez, C. Martínez-Cao, L. García-Alvarez, M.P. García-Portilla, P.A. Saiz, J. Bobes

**Affiliations:** 1 Psychiatry, SESPA Mental Health Services of Principado de Asturias, OVIEDO, Spain; 2 Department Of Psychiatry, University of Oviedo, Oviedo, Spain; 3 Neuroscience And Sense Organs, ISPA HEALTH RESEARCH INSTITUTE OF THE PRINCIPALITY OF ASTURIAS, Oviedo, Spain

**Keywords:** Depression, COVID-19, psychological impact, Age

## Abstract

**Introduction:**

COVID-19 pandemic and lockdown have provoked a considerable psychological impact in Spain. Some studies have reported greater psychological impact in the younger population. To date, no previous study has focused on depressive disorder (DD) patients based on their age.

**Objectives:**

To describe the psychological impact on DD according to age.

**Methods:**

Cross-sectional study of an online survey available from 19 to 26 March 2020. Out of a total of 21207 respondents, 608 (2.9%) reported suffering from DD (mean age ±SD = 41.2 years±14.07 [18-82], 80.6% women). The subsample (608) was divided according to age, “youngsters” <45 (57.4%)/ “elders” ≥45. DASS-21 and IES scales were employed. Statistical analyses: Chi-square, t-Student test.

**Results:**

Both groups did not differ (p>0.05) in sex, having COVID-19 symptoms, having family/friends infected, or income changes. While youngsters were single more frequently (68.8% vs 14.3%, χ² = 179.7, p<0.001), elders had somatic illness more frequently (64.8% vs 39.7% χ² =30.401, p<0.001). Youngsters obtained higher scores in depression (4.69 vs 4.1, T=5.413, p<0.001), anxiety (2.86 vs 1.97, T=5.249, p<0.001) and stress (4.48 vs 3.17, T=6.355, p<0.001) DASS-21 subscales, as in intrusive (3.42 vs 3.05, T=1.984, p=0.048) and avoidant (4.64 vs 4.11, T=3.056, p=0.002) IES scores.

**Conclusions:**

Despite the group of elders with depression being more vulnerable to severe COVID-19 disease and presenting more frequently somatic comorbidities, younger depressive patients suffered more from depressive, anxiety, stress and avoidant symptoms and intrusive thoughts, in line with previous reports in the general population.

**Disclosure:**

No significant relationships.

